# Effect of Solution Annealing on Fatigue Crack Propagation in the AISI 304L TRIP Steel

**DOI:** 10.3390/ma14061331

**Published:** 2021-03-10

**Authors:** Michal Jambor, Tomáš Vojtek, Pavel Pokorný, Miroslav Šmíd

**Affiliations:** 1 CEITEC IPM, Institute of Physics of Materials, Czech Academy of Sciences, Žižkova 22, 616 62 Brno, Czech Republic; jambor@ipm.cz (M.J.); smid@ipm.cz (M.Š.); 2 Institute of Physics of Materials, Czech Academy of Sciences, Žižkova 22, 616 62 Brno, Czech Republic; pokorny@ipm.cz; 3 Central European Institute of Technology (CEITEC), Brno University of Technology, Purkyňova 123, 612 00 Brno, Czech Republic

**Keywords:** solution annealing, fatigue crack propagation, martensitic transformation, crack closure, 304L steel, effective threshold, load shedding

## Abstract

Fatigue crack propagation in near-threshold regime was studied in the 304L austenitic stainless steel in two microstructural states: as-received (AR) with finer microstructure and low susceptibility to the transformation-induced plasticity (TRIP) effect, and solution-annealed (SA) with coarser microstructure and higher susceptibility to TRIP. At the load ratio *R* = 0.1 the threshold was higher in the SA state than in the AR state due to coarser grains and possibly the TRIP effect. In order to clarify the role of crack closure, experiments at *R* = 0.7 were done. The threshold in the SA state was still higher by 1 MPa·m^0.5^. This effect was identified as crack tip shielding induced by phase transformation, an example of a non-closure shielding effect. Higher resistance to crack growth in the SA state was attributed to promoted martensitic transformation in non-favorable oriented grain families rather than thicker martensite layers in the crack path area. The conclusions were verified by experiments at *R* = 0.7 and temperature 150 °C > M_s_ which did not reveal any notable difference in thresholds. However, the threshold values were affected by the load-shedding gradient *C* = −dΔK/da, which had to be equalized in both experimental setups inside and outside the furnace.

## 1. Introduction

Austenitic stainless steels are widely used engineering materials given their excellent combination of corrosion resistance and selected mechanical properties. They have applications in many industry sectors such as chemistry, energetic industry, food production and civil engineering. Due to the nature of the austenitic microstructure, these steels are also frequently used in welded structures [1,2,3]. Austenitic stainless steels are also characterized by excellent work hardening ability resulting in exceptional damage tolerance properties [4,5]. The work hardening characteristics can be further enhanced by carefully chosen chemical composition resulting in low stability of the austenitic structure responsible for the transformation-induced plasticity (TRIP) effect [6].

The TRIP phenomenon was frequently studied in the past [5,7,8,9,10,11,12]. The susceptibility of the materials to strain-induced martensitic transformation under external loading depends on a large number of external and internal factors. The important external factors affecting this process are temperature, type and magnitude of applied loading and strain rate [13,14]. The most important internal factor is the chemical composition which determines the stacking fault energy value. This material parameter controls deformation mechanisms which are active upon external loading [10,13,15,16,17]. Other internal factors affecting the susceptibility to the strain-induced phase transformation are the grain size, their distribution and initial dislocation structure [11,18]. Numerous studies showed that the TRIP effect in the austenitic stainless steel can significantly reduce the fatigue crack growth rate (FCGR) [8,9,19,20]. Several studies were carried out, in which the austenitic stainless steels were subjected to fatigue crack propagation tests at various temperatures with respect to the martensite-start (M_s_) temperature. Comparison of the FCGRs at equal stress intensity factor ranges ΔK for the initial martensitic and austenitic structure revealed significantly higher FCGR for the martensitic microstructure, suggesting that the martensitic transformation is the cause of lower FCGR in the metastable austenitic stainless steels rather than just the presence of the martensitic structure ahead of the crack tip [8,9].

Solution annealing is the standard heat treatment of the austenitic stainless steels. It aims to produce recovered microstructure after preceding cold working and primarily dissolve delta ferrite formed during primary solidification. However, in the light of the TRIP phenomenon, the dissolution of the delta ferrite increases chromium content in the adjacent regions, and together with grain coarsening, can enhance the material susceptibility to the martensitic transformation when externally loaded. Several studies have shown differences in the near threshold FCGR between cold worked and annealed austenitic stainless steels [8,9,19,20]. However, most of these studies were focused on different aspects of the TRIP and FCGR processes under variable conditions. Therefore, there are ambiguities about the direct role of the solution annealing on the fatigue crack growth properties and the magnitude of the effect on the obtained crack growth rates.

The presented study aims to evaluate the effect of the solution annealing of the AISI 304L metastable austenitic stainless steel on the fatigue crack propagation in the light of the strain-induced martensitic transformation phenomenon. Fatigue crack propagation tests were carried out at room temperature and at 150 °C, at which the strain-induced martensitic transformation is significantly reduced. The microstructure in the crack front vicinity, as well as the fracture surfaces, were examined using scanning electron microscopy (SEM). The obtained results indicate that the solution annealing results in the partial grain growth and the dissolution of delta ferrite colonies. These factors lowered the stability of the initial austenitic microstructure and promoted the strain-induced martensitic transformation in the solution-annealed state. Therefore, the resistance to fatigue crack propagation in terms of threshold and effective threshold of stress intensity factor range was studied in the solution-annealed state and compared to the as-received state.

## 2. Materials and Methods

### 2.1. Material

The investigated material AISI 304L steel was delivered in the form of hot-rolled sheet with the thickness of 20 mm. The as-received material state (AR) and the state after solution annealing (SA) were studied. The solution annealing at 1050 °C for 30 min was done in nitrogen protective atmosphere with subsequent cooling down by nitrogen flow. The heat treatment was applied on the specimens with final shape and dimensions. Chemical composition of the experimental material is shown in Table 1. The microstructure was examined by light microscopy. Samples were mechanically ground using sandpapers followed by the mechanical polishing with diamond abrasives. Subsequently, the sample surface was electropolished by a solution of 600 mL methanol, 360 mL ethylene glycol monobuthyl ether and 60 mL perchloric acid using a Struers Lectropol-5 device (Struers, Ballerup, Denmark). As the final step, the samples were etched using Beraha reagent. Documentation of the microstructure of the AR and SA states was carried out using the Olympus GX51 microscope (Olympus Corporation, Tokyo, Japan) equipped with a digital camera and polarized light contrast. The average grain size was determined using the equivalent diameter method. The delta ferrite content in both states was determined via magnetic induction measurements using Fisher Feritscope MP30 device (Helmut Fischer, Sindelfingen, Germany).

Standard tensile tests were performed using cylindrical specimens of the gauge length of 25 mm and the diameter of 8 mm. The tests were performed on the screw-driven universal testing machine Zwick Z50 (Zwick Roell GmbH, Ulm, Germany) at constant strain rate 0.002 s^−1^. Three specimens from both states were tested and the average values were calculated.

### 2.2. Fatigue Crack Propagation Tests

**Figure 1:**
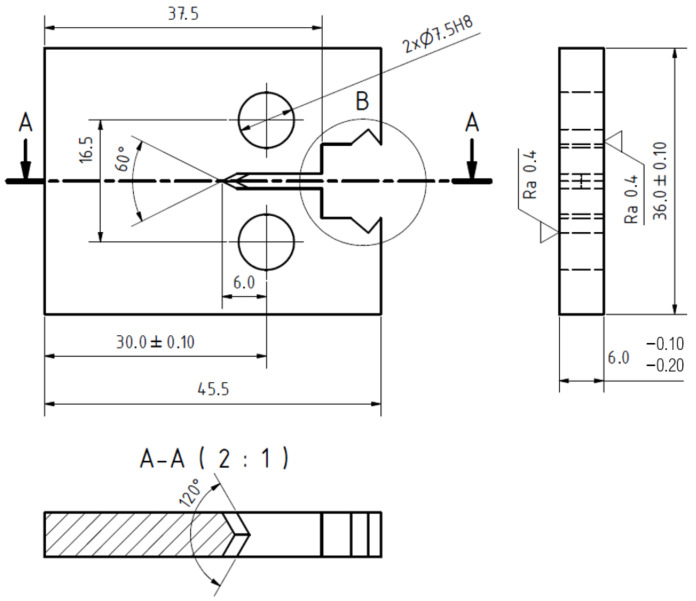
Geometry of the CT specimens used for the crack growth rate measurements.

The da/dN vs. ΔK curves were determined according to the standard ASTM E647 using the compact tension (CT) specimens with the parameter W = 30 mm and the thickness t = 6 mm, see Figure 1. The crack plane was oriented parallel to the rolling direction. The chevron notch root was machined to ensure symmetrical initiation of the fatigue crack with respect to both sides of specimen.

Measurement of the crack growth rates in terms of the da/dN vs. ΔK curves was carried out using the testing machine with a linear motor Instron e10000 (Instron, Norwood, MA, USA) and the resonant testing machine Zwick Vibrophore 25 (Zwick Roell GmbH, Ulm, Germany). In the case of Instron e10000 the capacity and maximal amplitude was 10 kN, while the frequency was kept at 70 Hz. The Zwick Vibrophore 25 possessed the loading capacity of 25 kN with the maximum amplitude of 12.5 kN. The loading frequency varied in the range from 60 Hz to 90 Hz, depending on the specimen stiffness. The experiments were done in a laboratory with controlled temperature and humidity. The temperature was set to 23 °C and the absolute humidity was kept at 10 g/m^3^ (the corresponding relative humidity is 50%). Additional tests were carried out in the environmental chamber Instron 3119-605 at elevated temperature 150 °C.

Crack propagation rates were determined at load ratios R = 0.1 and R = 0.7, where R is equal to K_min_/K_max_ and K_min_ and K_max_ are minimal and maximal stress intensity factors of the loading cycle, respectively. These load ratios were chosen to acquire representative da/dN vs. ΔK curves for the conditions with crack closure effects present (R = 0.1) and without crack closure (R = 0.7). Fatigue cracks were initiated in the notch by cyclic loading with a higher amplitude than the expected one for the threshold stress intensity factor range ΔK_th_. When a crack of 1 mm length was initiated on each side of the CT specimen, the load reduction method was started with the reduction of ΔK by 5% after each crack increment of approx. 0.1 mm. This procedure was kept until the crack was arrested at the loading then denoted as ΔK_th_.

For the tests performed at temperature 23 °C the crack lengths were measured optically using two digital Basler acA2040 cameras (Basler AG, Ahrensburg, Germany) equipped with the Azure-2514MM objectives. For the tests performed at the temperature 150 °C the crack opening displacement (COD) gauge Sandner EXR10-1o (Sandner Messtechnik GmbH, Biebesheim am Rhein, Germany) was used for determination of the crack length based on changes of the specimen stiffness. The stiffness for each crack length was computed from the upper quarter of the descending load-displacement curve in order to ensure that the crack was open. Calibration of the crack length measurement by the COD gauge was done using simultaneous optical and COD measurements at room temperature. In order to increase accuracy of the COD measurement at elevated temperature (150 °C), the specimens were removed from the environmental chamber after reaching the crack arrest at ΔK_th_ and they were observed in a light microscope. Based on the optical measurement, the crack length data obtained by the COD gauge were further calibrated. 

The crack propagation rate da/dN was estimated by Δa/ΔN, where Δa was the crack length increment in each step of the test with the number of cycles ΔN. The stress intensity factor was determined using Equation (1) according to [21]:

(1) $$\Delta K=\frac{\Delta P}{{\mathrm{tW}}^{\frac{1}{2}}}\cdot \frac{\left(2+\alpha \right)\left(0.886+4.64\alpha -13.32\alpha^{2}+14.72\alpha^{3}-5.6\alpha^{4}\right)}{{\left(1-\alpha \right)}^{\frac{3}{2}}}$$

where ΔP is the applied force range, *t* is the specimen thickness, W is the parameter of the CT specimen (W = 30 mm) and α is the ratio $\alpha =\frac{a}{W}$.

After the threshold was reached the specimens were examined to investigate microstructure in the vicinity of crack tip and crack path. The two side surfaces of the specimens were mechanically ground using a sandpaper followed by the electrolytic polishing using the Struers Lectropol device and the solution of 600 mL methanol, 360 mL ethylene glycol monobuthyl ether and 60 mL perchloric acid. During grinding a surface layer of material of at least 0.2 mm was removed to eliminate the free surface effects on crack propagation (character of the plastic zone and mechanism). The presence of the martensite in the crack vicinity could be monitored by various methods but, due to the localization of the phase transformation to small volume close to the fatigue crack, X-ray diffraction (XRD) measurements, as well as magnetic induction measurements, were not found to be feasible [22,23]. Therefore, the microstructural observations were carried out using a scanning electron microscope (SEM) Tescan Lyra 3 XMU (Tescan, Brno, Czech Republic), equipped with the backscattered electrons (BSE) detector and the electron backscattered diffraction (EBSD) camera.

## 3. Results and Discussion

### 3.1. Microstructure and Mechancal Properties

**Figure 2:**
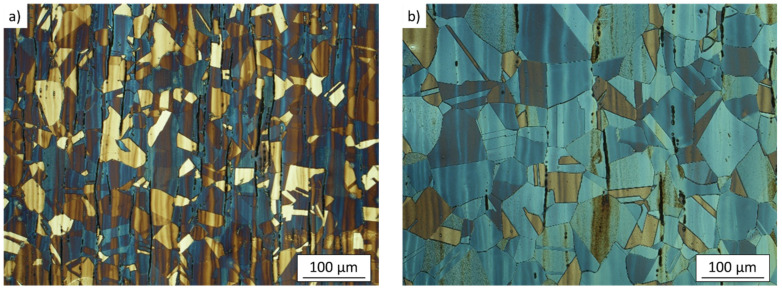
Microstructure of experimental material AISI 304L; (**a**) as-received state, (**b**) solution-annealed state. The rolling direction is oriented vertically. The dark stringers along the rolling direction are delta ferrite grains.

The microstructure in the AR state is shown in Figure 2a. It consists of equiaxed austenitic grains with frequent occurrence of annealing twins and delta ferrite stringers, formed along the rolling direction. The average grain size was measured to be 32 µm ± 11 µm excluding twin interfaces. The microstructure after SA is shown in Figure 2b. Similarly to the AR state, the microstructure consists of equiaxed austenitic grains with numerous annealing twins and delta ferrite stringers. The applied solution annealing treatment resulted in significant grain coarsening. The measured grain size was 48 µm ± 32 µm. The resulting structure was heterogeneous, containing zones of heavily coarsened grains and zones with the grains of almost initial size (mostly located between delta ferrite stringers which hinder grain boundary migration). Magnetic induction measurements revealed reduction of delta ferrite content due to solution annealing (1.24% in the AR state vs. 0.53% in the SA state). Martensite was not present in undeformed microstructure of both states.

**Figure 3:**
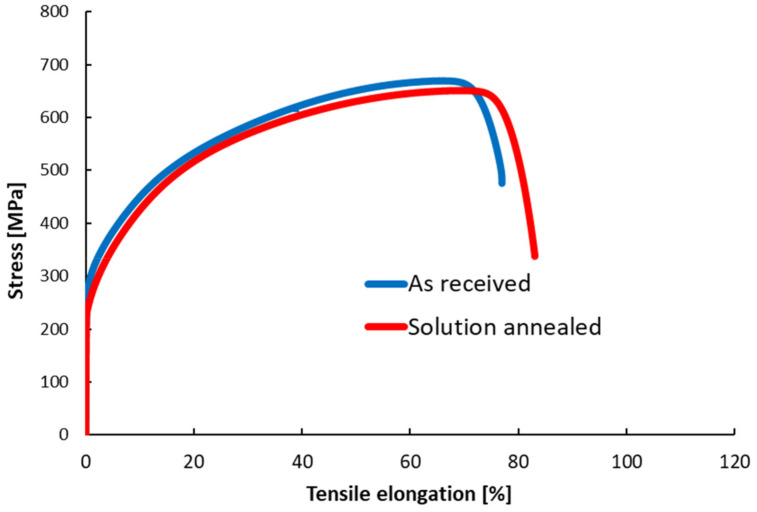
Stress-tensile elongation curves of experimental material AISI 304L.

The basic mechanical properties obtained from the tensile tests together with the corresponding stress-strain curves are shown in Table 2 and Figure 3, respectively. Subsequently, deformed microstructure after the tensile tests was examined. Cuts were prepared from the specimen gauge length with the cutting plane oriented along the specimen axis and the rolling direction. Careful positioning of the cuts within the gauge length secured exclusion of the necking area from observation. The cuts were subjected to the same preparation procedure as described above.

**Figure 4:**
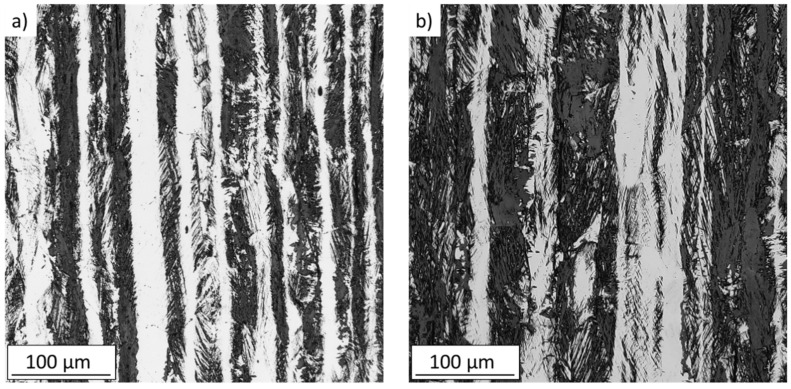
Microstructure of the tensile test specimens gauge lengths; the dark areas correspond to strain-induced α’-martensite, (**a**) as-received state, (**b**) solution-annealed state.

The comparison of obtained mechanical properties revealed differences in the stress-strain behavior after solution annealing. The applied heat treatment resulted in reduction of the yield strength, most likely due to reduction of a high dislocation density generated during the rolling process and due to a slight increase in the grain size. Tensile elongation of the SA state increased compared to the AR state (from 76% to 82%). Microstructure of the tensile specimens after the tests is shown in Figure 4. The post-mortem observation revealed increased volume fraction of the strain-induced martensite in the cross-section for the SA specimens. The determined volume fraction of the α’ martensite was 32.99% for AR state respectively 48.51% for SA state. The higher susceptibility to the γ-α’ phase transformation enhances the capability to accommodate larger amount of plastic deformation after solution annealing, resulting in the higher elongation at fracture.

### 3.2. Crack Growth Rates and Thresholds

**Figure 5:**
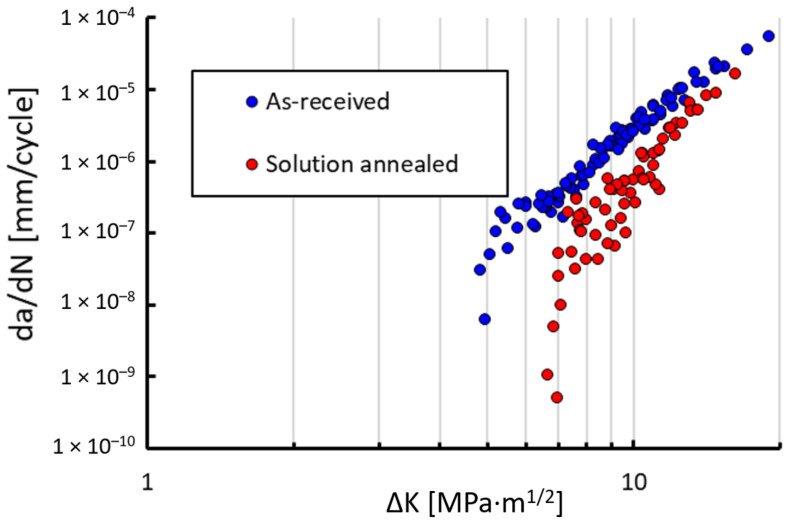
Measured crack growth rates da/dN at the load ratio R = 0.1 for the two microstructural states at room temperature.

**Figure 6:**
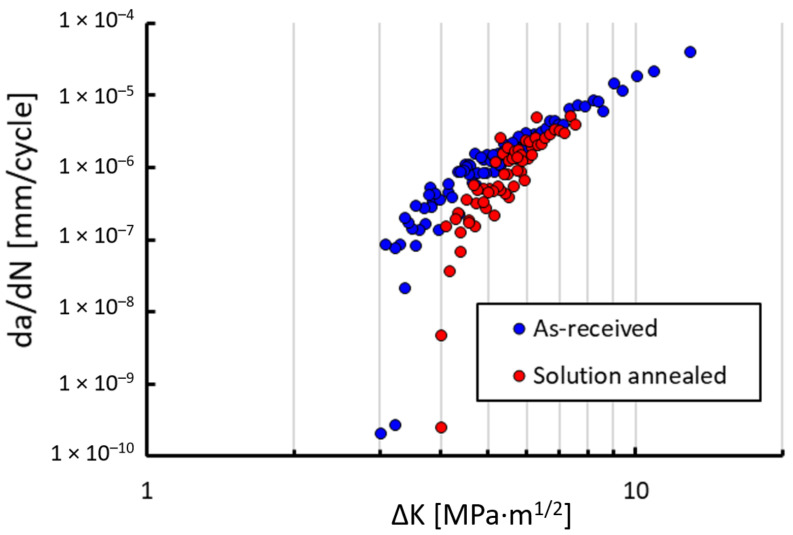
Measured crack growth rates da/dN at the load ratio R = 0.7 for the two microstructural states at room temperature.

Fatigue crack growth rates were measured using the CT specimens made of the 304L steel in two microstructural states, the as-received (AR) state and the solution-annealed (SA) state. Specimens fabricated in both orientations, parallel and perpendicular to the rolling texture, were investigated. However, no differences in crack growth behavior were detected and thus the results are presented regardless of these orientations. In Figure 5 the results from the tests at room temperature at the load ratio R = 0.1 are presented. A higher threshold of the SA material state resulted most likely from larger grains, which generally lead to higher plasticity- and roughness-induced crack closure [24]. Additionally, the more pronounced TRIP effect may have increased the threshold in the SA state. In order to make it more clear which mechanism was responsible for each effect and to what extent, the experiments at the load ratio R = 0.7 were performed, where crack closure should be suppressed. The diagram in Figure 6 shows the results of measurement at room temperature. There is still a difference between the thresholds, even at R = 0.7, which is quite unusual in steels, where the effective thresholds are almost always close to the value of about 2.5–3.0 MPa·m^0.5^. The threshold of the AR material state lies in this range. However, the SA material state had a higher threshold value of approx. 4 MPa·m^0.5^, which indicates that the enhanced TRIP effect was responsible for an increase of the threshold at R = 0.7 by approx. 1 MPa·m^0.5^.

**Figure 7:**
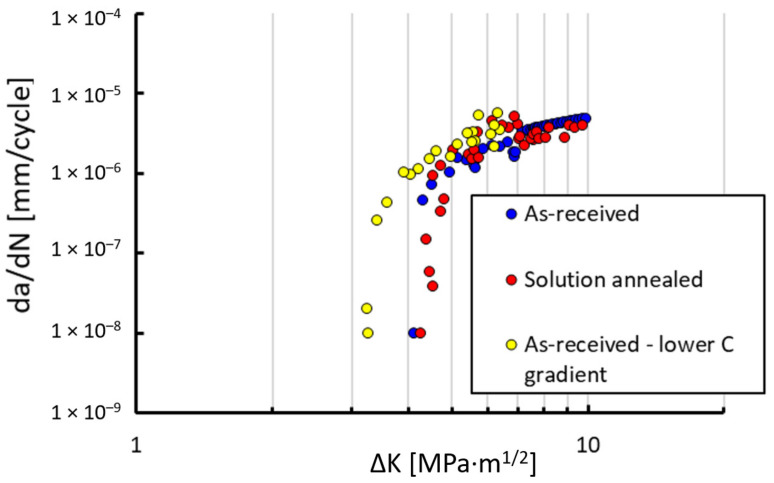
Measured crack growth rates da/dN at the load ratio R = 0.7 for the two microstructural states at the elevated temperature of 150 °C. The yellow points correspond to the experiment with a lower load-shedding gradient C = −dΔK/da.

In order to verify this effect, other experiments were done at the temperature of 150 °C, which is above the M_s_ temperature and where no TRIP effect should occur. The results are presented in Figure 7, where the blue points stand for the AR state and red points are for the SA state. In this case, the thresholds were approximately equal. However, both of them are somewhat higher than in the case of the specimen at room temperature with low TRIP effect. Due to difficulties with measurement of the crack growth rates inside the furnace, the conditions were not exactly the same as in the case of the specimens tested at room temperature. In particular, the gradient of load decreasing C = −dΔK/da was higher in the furnace. It is known that the *C* gradient affects threshold measurement under crack closure ratios (e.g., R = 0.1). However, as pointed out by Newman [25], the load shedding method can affect threshold values even at high load ratios. Therefore, an additional experiment was performed to verify the influence of the C gradient. The result of the test in 150 °C with a lower *C* gradient (close to that of the specimens at room temperature) are plotted in Figure 7 by the yellow points. It reveals that here the threshold is lower than in the case of a high *C* gradient and that the value is closer to the threshold for the AR state at room temperature (limited TRIP effect).

**Figure 8:**
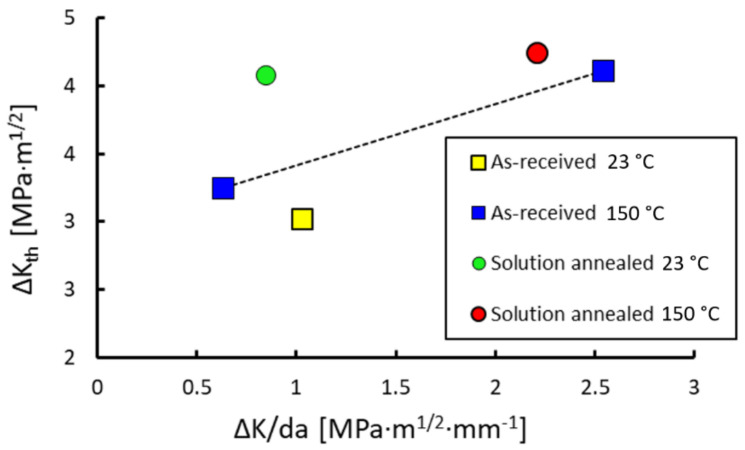
Influence of the load decreasing gradient C = −dΔK/da on the threshold value measured at the supposedly closure-free load ratio R = 0.7.

The influences of material state, temperature and the *C* gradient are summarized in Figure 8, where all measured threshold values for R = 0.7 are plotted. It shows that the strong TRIP effect in the SA material state led to a higher threshold compared to the AR state at room temperature. At the temperature of 150 °C > M_s_ the thresholds were equal for both states (suppressed TRIP effect in both of them), however faster load shedding resulted in higher thresholds. The additional test at 150 °C and the low C gradient confirmed this influence by revealing a lower threshold.

### 3.3. Microstructure in the Crack Tip Area

**Figure 9:**
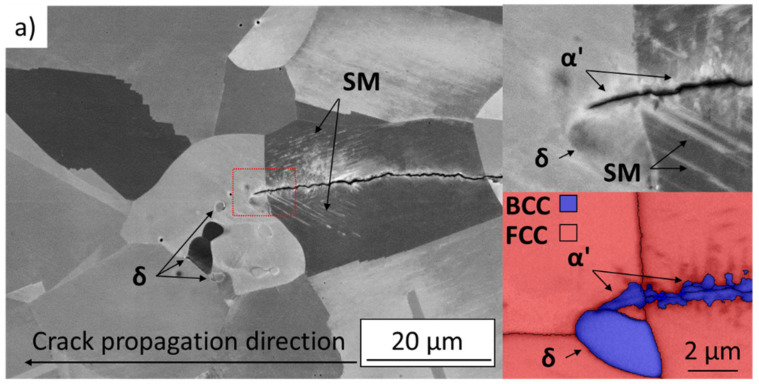

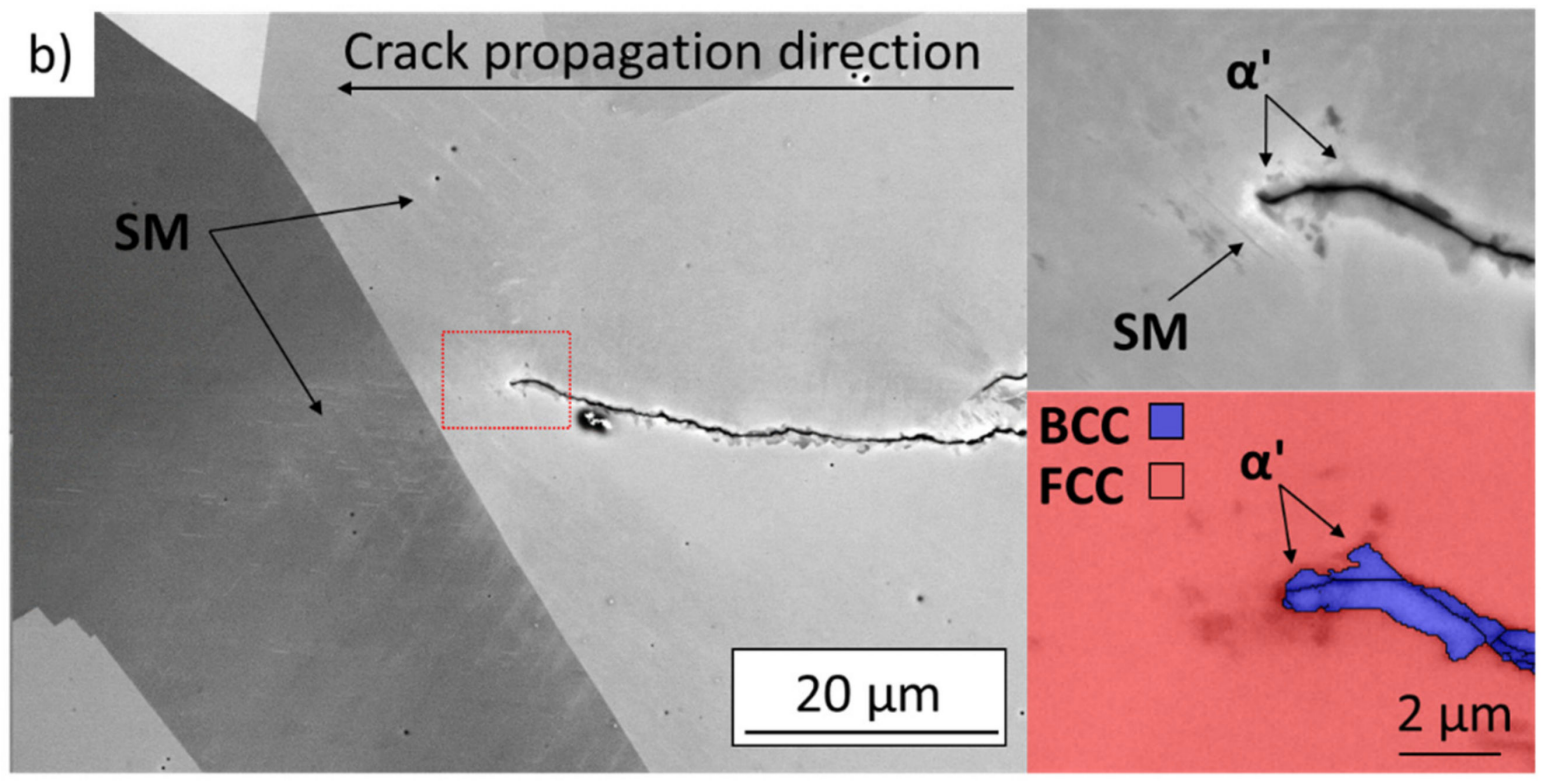
SEM images of the microstructure in the crack tip vicinity with the crack arrested at the threshold stress intensity factor, R = 0.7, 23 °C; (**a**) as-received state, (**b**) solution-annealed state. Detail of the areas marked by red rectangle is shown together with corresponding phase maps (acquired by EBSD).

Comparison of the microstructure in the crack tip vicinity is shown in Figure 9. The observed cracks were arrested at the threshold loading at room temperature. In both examined states, a continuous layer of the strain-induced martensite was present on each crack flank and ahead of the crack tip. The martensite layer thickness varied in grains with different crystallographic orientations, leading to difficulties in proper quantitative comparison. However, the maximal thickness of the martensite layer in the SA state appeared locally to be larger than in the AR state. The slip markings (SM) representing planar features of slip localization were observed in both states. The areas of intensive slip localization (e.g., in the detail of Figure 9b) can act as nucleation sites for the martensitic transformation [26,27]. The low contrast of the slip markings in Figure 9 could be explained by different orientation of the particular grain and the chosen imaging conditions, which were adjusted to optimize α’ martensite contrast. The crack path in both states is tortuous, suggesting numerous short-range crack deflections due to the ongoing phase transformation.

**Figure 10:**
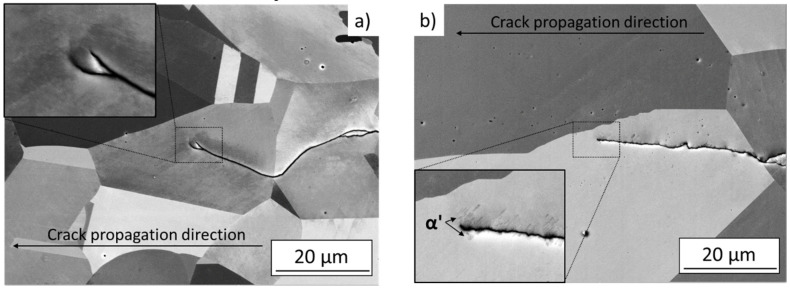
SEM images of the crack tip area after the crack arrest at threshold, R = 0.7, 150 °C; (**a**) as-received state, (**b**) solution-annealed state.

Elevated temperatures completely suppressed martensitic transformation in the AR state, while higher susceptibility to the TRIP effect in the SA state resulted in local presence of a small amount of martensite even after the tests at elevated temperature. The microstructures of the crack tip region after propagation at 150° C are shown in Figure 10. In this case, certain differences between AR and SA states were revealed. The examination was carried out stepwise with a material layer by layer removal by grinding to reveal the microstructure of the crack tip region in grains with various crystallographic orientations. The AR specimens did not reveal presence of any martensite. On the other hand, in the SA state local presence of the martensitic phase was identified (see Figure 10b). The amount of strain-induced martensite varied in grains with different orientations but no continuous layer of martensite was detected. The absence of continuous martensite layer resulted in a smooth crack path. Occasional short-range crack deflections were observed in the SA state, however, considering no differences in the obtained crack growth properties, it is supposed that this had negligible effect on the overall crack propagation behavior under given test conditions.

### 3.4. Fracture Surfaces

**Figure 11:**
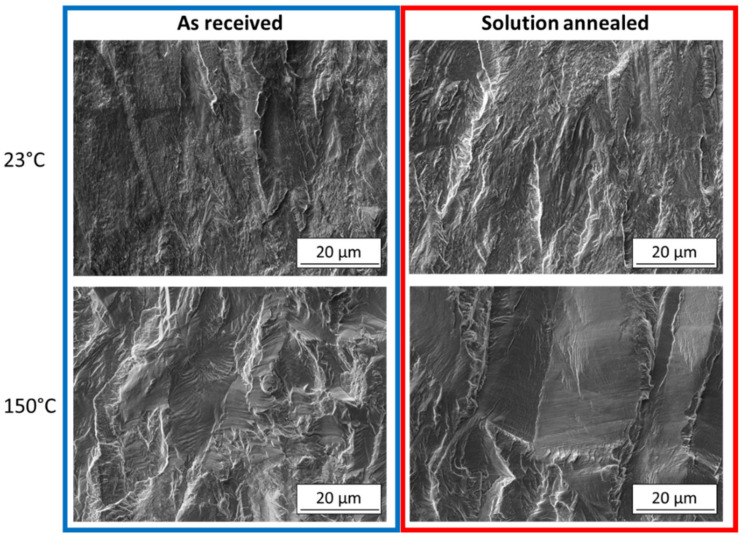
SEM images of fracture surfaces in the near-threshold growth area, R = 0.7.

Observation of the fracture surfaces supported the findings of the microstructural analysis. The SEM images of fracture surfaces from the near-threshold growth region are shown in Figure 11. At room temperature, no significant differences between AR and SA states are visible. The fracture surfaces have a quasi-cleavage character with poorly defined facets. Such fracture surface morphology was observed by other authors in case of fatigue crack propagation with the occurrence of strain-induced martensitic transformation [8,20,28]. On the other hand, the fracture surfaces from the experiment at 150 °C exhibited faceted character, suggesting absence of the notable martensitic transformation, while planar fatigue crack propagation was dominant. A large difference in the grain sizes within the observed region is clearly visible.

### 3.5. Discussion

Microstructural observations did not reveal significant increase of the average martensite layer thickness in the SA state. The effect of enhanced strain-induced martensite transformation in the case of the SA state can thus be attributed to promotion of martensite formation also in the grains with non-favorable orientation for the transformation, which did not occur in the AR state. During solution annealing, material undergoes notable changes in chemical heterogeneity due to diffusion processes resulting in a decrease of austenite stability [29]. Considering the crack front in three dimensions, the crack passes through large number of grains of various crystallographic orientations at the same time. Therefore, the facilitation of the strain-induced martensite transformation in the non-favorable oriented grain families could decrease the overall crack driving force and increase the fatigue crack growth resistance at low and medium loading levels. 

In the SA state, occasional presence of the strain-induced martensite after the tests at 150 °C was observed, which is in contrast with the total absence of martensite in the AR state. However, the fatigue crack growth tests revealed the same behavior for both states under given conditions, suggesting that the occasional occurrence of the martensitic transformation in the crack tip region did not affect the macroscopic crack growth properties. For the TRIP phenomenon to have any effect on fatigue crack growth, a continuous martensite layer has to be formed, as was revealed in the tests performed at room temperature.

## 4. Conclusions

In this work the 304L austenitic stainless steel in two microstructural states was characterized from the point of view of resistance to fatigue crack propagation. The first microstructural state was the as-received (AR) state with fine microstructure and low susceptibility to the TRIP effect, while the second one was the solution-annealed (SA) state with coarser microstructure and higher susceptibility to the TRIP effect. The tests at the load ratio R = 0.1 revealed higher threshold value in the SA state than in the AR state, which was at least partially caused by coarser microstructure leading to higher levels of plasticity- and roughness-induced crack closure. In order to separate the mechanisms of crack closure and the non-closure effect of TRIP on resistance to fatigue crack growth, experiments at the load ratio R = 0.7 were done. Here, the threshold in the SA state was still higher by 1 MPa·m^0.5^ than in the AR state. The crack tip shielding induced by phase transformation was suggested to be responsible for this difference.

Microstructural observation did not reveal significant differences in occurrence of martensitic layers in the crack tip area in the SA and AR states. This indicates that the increased fatigue crack growth resistance of the SA state was most likely caused by promotion of phase transformation in the non-favorable oriented grain families rather than in the overall increase of the martensite layer thickness.

To further support the idea of the influence of TRIP on threshold at R = 0.7, tests at the temperature of 150 °C > M_s_ were done. In this case, the thresholds were approximately equal in both microstructural states. However, it was also found that the load-shedding gradient C = −dΔK/da influenced the measured threshold values, which were higher in the case of a higher C gradient. Such effect is traditionally considered only for the crack closure load ratios. In order to have valid results for comparison of the thresholds, it is necessary to maintain equal C gradients in all crack growth measurements. Differences in the C gradient resulted from different testing methodologies inside the furnace and in free air at room temperature. Once the C gradient of the elevated temperature experiments was equalized to that of the tests at room temperature, the threshold was in agreement with that of the AR state at room temperature (low TRIP effect).

No continuous martensite layer was detected at the crack path area after the tests at 150 °C. In the AR state, martensite was fully absent, while in the SA state, local martensite islands were present. Similar fatigue crack propagation properties for both states at 150 °C suggested that local martensite formations in the SA state had minor effect on the overall fatigue crack growth properties. Therefore, in order to have notable effect of the strain-induced martensitic transformation on fatigue crack propagation, a continuous martensite layer has to be developed.

## Figures and Tables

**Table 1:** Chemical composition of experimental material AISI 304L (in wt. %, provided by supplier).

C	Cr	Ni	Mn	S	P	Si	Fe
0.023	18.12	8.18	1.79	0.003	0.04	0.17	Bal

**Table 2:** Basic mechanical properties of both states of the experimental material AISI 304L.

AISI 304L	0.2% Offset Yield Strength [MPa]	Ultimate Tensile Strength [MPa]	Elongation at Fracture [%]
As-received state	266.6 ± 1.8	665 ± 4.2	76.5 ± 0.5
Solution-annealed state	234.1 ± 2.1	650.1 ± 1.5	82 ± 1.7

## Data Availability

The data presented in this study are available on request from the corresponding author.
